# *“I’ve accepted it because at the end of the day there is nothing*, *I can do about it”*: A qualitative study exploring the experiences of women living with the HIV, intimate partner violence and mental health syndemic in Mpumalanga, South Africa

**DOI:** 10.1371/journal.pgph.0002588

**Published:** 2024-05-06

**Authors:** Mpho Silima, Nicola Christofides, Hannabeth Franchino-Olsen, Nataly Woollett, Franziska Meinck

**Affiliations:** 1 School of Public Health, University of the Witwatersrand, Johannesburg, South Africa; 2 Division of Health Behavior and Health Promotion, College of Public Health, The Ohio State University, Columbus, Ohio, United States of America; 3 Department of Visual Arts, University of Johannesburg, Johannesburg, South Africa; 4 School of Social and Political Sciences, University of Edinburgh, Edinburgh, United Kingdom; 5 School of Health Sciences, North-West University, Vanderbijlpark, South Africa; NYU Grossman School of Medicine: New York University School of Medicine, UNITED STATES

## Abstract

In South Africa, Mental Health (MH), HIV, and Intimate Partner Violence (IPV) form a syndemic, that disproportionately affects women. These challenges are often co-occurring and create complex adversities for women. Recognising these intersections and the broader socio-cultural dynamics at play is crucial to understanding the layered experiences of these women and developing effective interventions. This research explores the experiences of the women living with at least two of the epidemics (HIV, IPV and or MH) and how they cope. A qualitative study design was used and 20 women (22–60 years) were recruited from Mpumalanga, South Africa. To be eligible for the study the women had to have experienced at least two of the epidemics. Data were collected through home-based interviews, arts-based activities, and analysed thematically using MAXQDA (2022) software. MH challenges were prevalent among all the participants and were linked to both IPV and HIV, resulting in symptoms such as anxiety, depression, and suicidal thoughts. In relation to the HIV-MH link, MH challenges in this combination included feelings of denial, sadness and anxiety related to participant’s HIV diagnosis. A bidirectional relationship also existed in the IPV-MH group where pre-existing MH challenges among women increased their vulnerability of having violent partners, whilst IPV also increased MH challenges. In the IPV-MH-HIV group early childhood violence exposure was linked with MH challenges and later victimization and vulnerability to HIV. Participants primarily used religion, acceptance, occasional alcohol, and family support as coping strategies. Particularly in IPV situations, alcohol use/misuse was the most prevalent coping strategies. The study highlights the syndemic relationship between HIV, IPV and MH challenges among South African women living in a peri-urban community, with a central emphasis on MH challenges. Interventions should holistically address these challenges, with particular focus on MH challenges, cultural sensitivity, and promotion of healthy coping strategies.

## Introduction

Women living with HIV or AIDS (hereafter referred to as HIV for brevity) and experiencing intimate partner violence (IPV) often face various mental health (MH) challenges. Research indicates that there is a higher prevalence of MH disorders among people living with HIV. Specifically, conditions such as depression, post-traumatic stress disorder (PTSD), anxiety, psychosis, and alcohol misuse are observed, which are often attributed to psychological stress, antiretroviral medication side effects, social stigma, discrimination, and lifestyle factors [1, 2]. Particularly, women living with HIV are also more likely to experience coexisting MH conditions and exhibit poorer overall MH than men [3]. The increased vulnerability is highlighted in established research which indicates existing gender disparities in MH conditions such as depression [4] anxiety [5] and PTSD [6, 7]. Furthermore, in the context of HIV these gender disparities in MH conditions may further be exacerbated [8]. Complicating matters further, IPV contributes to adverse MH outcomes, including anxiety, depression, PTSD, and increased suicide risk [3]. The primary mechanism that links IPV to the latter MH outcomes is IPV-induced traumatic stress [9, 10]. Moreover research also shows that IPV and MH disorders often have a reciprocal relationship, where one can increase the risk of occurrence of the other [9, 10]. The compromised cognitive and emotional resources that often accompanies MH disorders can make individuals more vulnerable to manipulative or abusive behaviors, thereby increasing their risk of becoming victims of IPV [11]. Previous research has also established a reciprocal link between IPV and HIV, indicating that those subjected to IPV have a 48% higher risk of HIV infection compared to their counterparts who haven’t faced such violence. The heightened risk can be attributed to factors such as coercive sex by an abusive partner and their propensity for engaging in risky sexual behaviours and reduced condom use [12].

The interconnectedness between HIV, IPV and MH disorders can be better understood using syndemic theory as proposed by Merril Singer [13]. Singer defines a syndemic as the complex interplay existing between different epidemics, especially in the context of social inequality and health disparities [13]. While SAVA primarily focused on the intersection of substance abuse, violence, and AIDS, our study expands this to encompass a broader range of MH challenges. HIV has long been long been recognized as an epidemic globally [14, 15], however, we argue that IPV and MH conditions can also be categorized as epidemics. In South Africa IPV is a significant public health issue, ranking as the second highest burden of disease after HIV or AIDS. It also contributes substantially to disability adjusted years (DALYs) especially among women where IPV and child sexual abuse are common [16]. In terms of MH conditions, there has been a significant global increase, with a 13% increase over the past decade. Additionally, these conditions are now responsible for one in every five years lived with a disability [17]. These trends highlight the epidemic nature of both IPV and MH.

The emphasis of the syndemic theory is that epidemics do not exist in isolation, but rather are often interconnected and co-occur or cluster in social groups as a result of harmful social conditions such as poverty and stigma. These conditions often interact in ways that contribute to an increased overall burden of disease. The Substance Abuse, Violence and AIDS (SAVA) syndemic was first identified by Singer, he argued that contextual factors such as poverty, inadequate housing, family instability, drug related violence worked in conjunction to exacerbate the conditions of SAVA simultaneously [13]. Much research has been conducted on the SAVA syndemic, yet there is still very limited data on the HIV, IPV and MH as a syndemic. As shown, the intersection of HIV, IPV, and MH disorders can exacerbate the health outcomes associated with these conditions.

### Coping strategies of women with the HIV, MH and IPV syndemic

Understanding how women living with the HIV-IPV-MH syndemic cope in the face of these intertwined epidemics is critical. Research has shown that the coping strategies used by people living with HIV and those experiencing IPV have a link to the emergence of MH consequences [18, 19]. Therefore, exploring the coping strategies of these women could highlight important strategies that have the potential to protect their psychological and physical wellbeing. Research shows that psychological reframing of negative events and social support have proven essential for women experiencing MH and HIV management, while acceptance and hope serve as a popular coping strategies for all three epidemics [20–23]. Furthermore, adhering to HIV treatment has been found to be a key coping strategy for women living with HIV, largely driven by their sense of responsibility to their family and in particular their children [21]. In the IPV context, coping strategies are more diverse, according to a systematic review of US studies, it was found that religious or spiritual reliance and active abuse resistance were common whilst self-criticism and substance misuse were less common [24]. Contrary to this, a systematic review of South African studies found that many of the women who had experienced IPV used avoidance and distraction as a way to cope and this included substance abuse/misuse [25]. Seeking help has also been found to be a coping strategy among some IPV survivors, however this varies and is dependent on the severity of the victimization as well as resources available to the woman [24]. A comprehensive understanding of the root causes that lead to specific coping strategies, can help us in developing interventions that can target specific issues so as to activate more adaptive coping strategies. Identifying coping strategies of people living with HIV, IPV and MH syndemic has important implications for healthcare providers and can lead to a more holistic approach to managing the epidemics.

## Present study

The primary objective of the present study was to explore the experiences of women navigating life with the intersections of HIV, IPV and MH challenges in a peri-urban community in Mpumalanga South Africa. We aimed to analyse the interplay of the epidemics and better understand the coping strategies used by the women. Furthermore, we sought to identify variations in coping strategies based on the different combinations of the epidemics experienced by the women.

## Methods

### Study design

To answer the study objectives a qualitative study design was used. The use of this approach was important in that it helped in exploring the research aims in more depth. We were able to have a deeper understanding of the participants experiences, perspectives and attitudes in relation to living with the HIV, IPV and MH syndemic. Furthermore, using a narrative approach helped to emphasize the personal experiences of the participants which in turn allowed for their individual stories to take centre stage. By focusing on these narratives, we were able to attain rich, detailed data and as a result enhance the depth of the research findings. A narrative inquiry is a type of qualitative research method that uses the stories of the participants as the primary source of data [26]. The methodology has been used across various fields to explore culture, history, identity and people’s lifestyles through the narrators’ experiences [27]. In particular the approach includes investigating human experiences through narratives or producing the data in a narrative format [28].

### Study setting

The current study was conducted in a peri-urban area within the Enhlanzeni District Municipality of Mpumalanga Province, South Africa from October 2022 to February 2023. Mpumalanga province has an estimated population of approximately 4 million people [29], with the primary languages being spoken being siSwati, isiZulu, Xitsonga and isiNdebele [30]. The study site is often categorized as a township, and primarily consists of brick houses. Common challenges in the community include inconsistent supply of basic services such as water and electricity with much of the infrastructure being informal. Unemployment is also rife, with the most common forms employment being domestic work, bricklaying and farming. The HIV prevalence in the district is reported to be 19.% among the adult population [29, 31]. In terms of IPV, 28% of women in Mpumalanga have reported experiencing physical abuse from their current partner at some point in their lives [32]. Moreover, a national study found that 29,2% of adults in Mpumalanga indicated that they had experienced common mental disorders at some point in their lives lifetime [33].

### Sample

Twenty participants were purposively selected for the study. Selection of the participants was based on their responses to specific questionnaire items from the Interrupt_Violence Study [34]. Participants were between the ages of 20–60 years and had to screen positive for HIV, IPV and/or MH challenges. Participants did not have to have experienced all three epidemics to be included in the study; any combination of the two epidemics was sufficient for inclusion.

During the quantitative interview, there were automatic referral flags that were produced if a participant disclosed behaviours, thoughts, or feelings of concern (S1 Table). The Flags were used to create voluntary and mandatory referrals to the study social worker. To identify participants who had experienced at least two of the epidemics, we noted participants with two or more flags for the epidemics of interest, indicating they had self-reported some combination of MH, IPV, and HIV. Thereafter we did a detailed analysis of the participants’ responses to the individual items for MH, IPV and HIV in the questionnaire. The participants were categorised into groups based on their questionnaire responses. In terms of MH challenges participants answered questions in the following screening tools: National Institute for Mental Health’s Ask Suicide Screening Questions (ASQ) for suicidality risk [35], Generalized Anxiety Disorder Screener for adult for anxiety [36], Patient Health Questionnaire for depression [37] and Post Traumatic Stress Disorder-8 (PTSD-8) for PTSD [38]. For IPV experience, participants needed to have answered yes to any of the questions in the WHO instrument for physical and sexual violence experience [39]. HIV status was determined either through self-reporting or identified during the voluntary HIV test administered by the interviewer.

Furthermore, the participants in the study were either mothers or primary caregivers for a child or children aged 17 or younger at the time of enrolment. This was because the data from the current study stems from a larger study which focussed on the experiences of parenting a child under the age of 17.

### Data collection tools and procedure

The lead author alongside two research assistants conducted in-depth interviews which lasted between 60 to 90 minutes with each participant during October 2022-February 2023. The interviews were conducted in the participants’ preferred languages of SiSwati or Xitsonga and took place face-to-face in a private space at a time convenient for the participants. The interviews allowed for probing into participants’ experiences with IPV, living with HIV, and navigating MH challenges. We explored their past and current experiences with IPV, the impacts of HIV diagnosis and its disclosure, and the community’s potential stigma towards HIV. Furthermore, their MH journey was explored in the context of these intersecting epidemics. For more details on the interview guide see S1 Text.

To stimulate rich conversation, we used arts-based techniques such as the Kinetic Family Drawing (KFD), River of Life, and Sandbox [40–42]. Based on the depth of data obtained from the interview, some participants were asked to participate in more than one activity, which contributed to the data’s richness. The probes used for the arts-based techniques can be found in S2 Text. The decision regarding which participants engaged in more than one activity was participant driven, in some cases where a participant had challenges with an activity, we switched to another activity. Conversely if a participant engaged well with one activity and this provided rich data, a second activity was used to help organize the story more effectively and to explore deeper into specific events.

With the KFD, participants drew two pictures representing their family of origin and current family dynamics, helping them to recall memories and build rapport with the interviewer [40, 43]. In the River of Life activity, participants visually drew their life journey, showing significant positive and negative events [41]. The Sandbox activity, inspired by sandplay therapy, provided participants a with medium to create three-dimensional scenes, that facilitated an artistic narrative of their experiences [42, 44]. To capture the story, multiple photos were taken of the participants’ sandbox from various angles and at different times especially when participants changed the positioning of the figures during the interviews. The participants were also audio recorded while they were describing the scenes in the sandbox. This allowed us to capture the story as it unfolded.

### Ethical considerations

The study received ethical clearance from the Human Research Ethics Committee (HREC) of the University of the Witwatersrand (M220526), ensuring adherence to recognized ethical standards for research. The main Interrupt Violence study was also ethically approved (M190949) by HREC, University of Edinburgh (No: 264227) and Provincial Department of Health Mpumalanga (MP-202012–003). All participants provided voluntarily written informed consent to participate and to have the interview audio recorded. The consent forms were distributed and explained to the participant prior to data collection in their preferred language, and they clearly outlined the objectives of the study, the nature of the participant’s involvement, and how the collected data would be used and stored to maintain confidentiality. Participants were also informed of their right to withdraw from the study at any time without any repercussions. To protect participants’ confidentiality, unique, non-personally identifying ID numbers and pseudonyms replaced participants’ real names in all research materials. Photographs were taken of participants’ artwork, with their explicit consent, to supplement data analysis. All data, including these photographs, were securely stored. Recognizing the sensitive nature of the study and the potential for psychological risk, we implemented a distress protocol. This protocol guided the interviewer in responding sensitively to a participants’ emotions during and after the interview, ensuring timely provision of follow-up resources and support. A full-time social worker from the Interrupt_Violence study was also readily available for necessary or mandated referrals. We had 10 mandatory referrals; 5 non mandatory referrals and 5 participants did not require a referral. Mandatory referrals included cases such as participants experiences of domestic violence including a weapon, suicidality symptomology especially in situations where there was a child in the home. Non- mandatory referrals included any instances where the participant felt that they would benefit from speaking to the social worker, this ranged from issues regarding accessing their social grants, accessing psychological assistance for their MH challenges and challenges of food insecurities.

### Data management and analysis

All interviews were translated, transcribed, and analysed using Braun & Clarke’s six-step thematic analysis [45]. The initial coding framework was deductively developed based on the study’s objectives and an early review of transcripts [46]. Subsequently, a codebook was developed, which included the coding structure used to represent different themes and patterns found in the data.

The research team then applied the codes using several transcripts, ensuring they were relevant and appropriate to capture the data and that they captured the study’s focus. For this analysis, we used MaxQDA 2022 software. Furthermore, an initial six transcripts were reviewed collectively by the research team to ensure the collected data’s depth and richness. This exercise helped us to fine-tune our exploration areas, identify points requiring clarity, and identify any emerging themes [47, 48].

## Findings

### Characteristics of participants

The participants in the study were grouped into three categories based on their experiences with HIV, IPV and MH challenges. The first group consisted of seven participants, whose ages ranged from 24 to 45 years. Each participant in this group had experienced or were living with all three of the epidemics as disclosed in the questionnaire (HIV-IPV-MH syndemic). The second category included eight participants between the ages of 23 and 57. These individuals had experienced the HIV-MH syndemic. Lastly, the third group had five participants, aged between 22 and 49, who were living with the IPV-MH syndemic. For more detailed specifics on each of the participants refer to Table 1 below. Pseudonyms have been used to conceal the identities of the participants.

**Table 1:** Participant characteristics and epidemic combinations.

Participant	Age	Number of Children	Relationship Status	Education Level	Employment Status	Additional Information
**Epidemic combination: HIV, MH and IPV**						
**Noxolo**	28	Three	In a relationship	Grade 7	Unemployed	Noxolo is currently experiencing violence with the current partner. She shared MH challenges, with symptoms linked to depression and anxiety
**Diana**	43	One	In a relationship	Grade 1	Unemployed	Diana experienced IPV in the past with the current partner, although at the time of the interview the violence had ceased. She shared MH challenges with symptoms linked to depression.
**Silindile**	38	One	In a relationship	Grade 10	Part-time employment	Silindile’s experiences of IPV were from a previous partner. She shared MH challenges with symptoms linked to suicidality.
**Nokuthula**	26	Two	In a relationship	Grade 11	Unemployed	Nokuthula’s experiences of IPV were from a previous partner, the father of her first child. She shared MH challenges with symptoms linked to depression.
**Sphe**	28	Two	In a relationship	Grade 12	Unemployed	Sphe’s was in a violent relationship at the time of the interview. She shared MH challenges with symptoms linked to depression.
**Lerato**	24	One	In a relationship	Grade 8	Unemployed	Lerato’s experience of IPV is with her previous partner. She shared MH challenges with symptoms linked to suicidality and depression.
**Paula**	45	Three	In a relationship	Grade 6	Unemployed	Paula has a history of violence with the current partner However, at the time of interview the violence had ceased. She shared MH challenges with symptoms linked to depression
**Epidemic combination: HIV and MH**						
**Nono (CG 7379)**	41	Four	Widowed and single	No schooling	Part-Time Employment	Nono shared MH challenges with symptoms linked to anxiety and suicidality.
**Rejoice**	25	One	In a relationship	Grade 12	Casual Work	Rejoice shared MH challenges with symptoms linked to depression and anxiety.
**Xoli**	23	Three	Single	Grade 11	Unemployed	Xoli shared MH challenges with symptoms linked to depression and anxiety.
**Ntombi**	26	One	In a relationship	Grade 12	Part-time employment	Ntombi shared MH Challenges with symptoms linked to depression, she was also clinically diagnosed with Trichophagia
**Phindi**	48	Five	Married	Grade 12	Unemployed	Phindi shared MH challenges with symptoms linked to suicidality.
**Pauline**	42	Three	In a relationship	Grade 8	Full-time employment	Pauline shared MH challenges with symptoms linked to depression
**Nelly**	57	Two	Single	Grade 6	Unemployed	Nelly shared MH challenges with symptoms linked to depression and anxiety
**Adelaide**	46	Five	Married	Grade 7	Part-time work	Adelaide shared MH challenges with symptoms linked to suicidality and anxiety.
**Epidemic combination: IPV and MH**						
**Palesa**	22	Two	Single	Engineering Diploma	Unemployed	Palesa is caregiving for her sister’s children. At the time of the interview, she was not in a violent relationship, her history of IPV is from previous partners. She shared MH challenges with symptoms linked to depression.
**Nomie**	28	Two	In a relationship	Grade 5	Unemployed	Nomie was in a violent relationship at the time of the interview. She shared MH symptoms linked to depression.
**Cindy**	49	Two	Married	Grade 8	Unemployed	Cindy has a history of violence with her current partner, however at the time of the interviewer the violence had ceased. She shared MH symptoms linked to depression and anxiety
**Londiwe**	42	Three	Married	No schooling	Part-time employment	Londiwe has a history of violence with her current partner, however at the time of the interviewer the violence had ceased. She shared MH challenges with symptoms linked to suicidality and depression.
**Ntomfuthi**	28	One	Single	Grade 12	Part-time employment	Ntomfuthi has a history of violence with her previous partner, however at the time of the interview she was single. She shared MH challenges with symptoms linked to depression.

### Intersecting epidemics and participant’s experiences

Our study found that the interaction between the epidemics often created a mutually reinforcing dynamic that escalated the overall burden on the individuals affected.

### Experiences of living with HIV-MH syndemic

Our findings highlighted a significant relationship between MH challenges and HIV. An exploration of the women’s experiences revealed that an HIV diagnosis often triggered feelings of denial and a struggle with accepting their status for instance, when Nono (41) was asked to share how she felt when she was first diagnosed with HIV, she responded:

*“It was very painful to even accept it*, *but I had to accept it and let it pass*. *But to be honest I only really started to accept and get healing maybe six years after I was diagnosed*. *You know it’s hard when you know all the sexual partners you have had in your life*, *for me it was only two people so it hurt for this to happen to me*. *But it was only after I was positive that people told me about his history*, *it was then that I heard of all his past sexual partners who had died from HIV*. *When I found out I was always crying*, *constantly crying*. *Asking myself for how long I will I have to suffer like this*.*”*.

Furthermore, for many of these women, an HIV diagnosis triggered symptoms linked to depression, anxiety, and in extreme cases, suicidal ideation. Ntombi, (26), conveyed the anxieties accompanying her HIV positive status,

*“It stresses me when*, *I think of what will happen when he [son] is grown up*. *I worry a lot especially when I think of times when I will not be around anymore*. *My heart is sore and I’m constantly scared*.*"*

For those participants who reported suicidal ideation, it was often in situations where their children had also acquired HIV. Silindile’s (38) son, for example, was born with HIV, when asked which of all of the challenges, she had encountered is the most difficult for her to accept, she responded:

*“I would say the most difficult for me is the HIV status because it directly affects my son*. *I don’t even know where to begin with solving that situation*. *How will I ever tell my son*, *he won’t even understand how he contracted this virus*. *This is the thing that breaks my heart the most*, *even now*, *sometimes I just have these feelings of wishing I could kill myself*. *Every time I think about his father*, *I just wish I had never met that man*, *if it were up to me*, *I would have never even walked in the same direction as that man*.*”*

Additionally, the perceived burden and the accompanied denial of an HIV diagnosis often significantly impacted their decision-making regarding treatment initiation. Paula, (45), shared how her HIV status and the emotional toll it took delayed her initiation of treatment:

*“I wasn’t ready to accept*… *so I waited five years after being diagnosed*, *I felt I needed to counsel myself before I could start taking treatment*. *There was a time where I did something*… *I would go fetch pills at the clinic and throw them away afterwards*, *then I fell sick*. ***Interviewer*:** *“What happened afterwards*?*”* ***Paula*:** *“I do not remember exactly who it was*, *maybe it was the nurse*, *they told me not to stop taking the pills*. *They asked me who I want to leave my children with*. *I went back to the clinic and I never stopped taking them*.*”*

### Experiences of living with IPV-MH syndemic

For the participants who had experienced IPV, we found that many of them also reported symptoms indicative of anxiety and depression. Cindy (49) had been in an abusive relationship with the father of her children for many years, she shared how being in this relationship put her in a constant state of fear and worry:

***Interviewer***: *“So*, *were there other times in your life where you felt constantly scared or worried*?*”* ***Cindy*: *“****Yes*, *it was during that time of the abuse*. *I would worry all the time about what will happen to my children if I leave*. *I would look around at other people’s lives and feel like I was the only one going through this and I didn’t understand why my life was the way that it was*. *I was so afraid of this man; I thought one day he will kill me*.*”*

Furthermore, Palesa (22), detailed how her abusive relationship created a constant state of anger in her, which she unconsciously directed towards her son. She shared:

*"It was difficult*, *I won’t lie*, *I was angry*, *I was an angry mother*, *very angry*. *Even when he [son] would try and speak to me*, *I would just insult him to the point that I would even regret it afterwards*. *I would get pissed off by my partner and take it out on the closest person to me and usually*, *it was my son he would do a small thing*, *like spill water*, *and I would overreact*. *Like*, *I realised that the way I was hitting him was not normal*. *I would take out a lot of pain on him*, *but I learned that he doesn’t know anything*, *he is a baby”*.

On the other hand, some participants reported the relationship between MH and IPV as bidirectional where they felt that their MH challenges also lead to violence in their relationship. Nomie (28) shared how her challenges with anger which spurred from her childhood trauma often impacted on her intimate relationships at times leading to violence:

*“I’m one person who is quick to anger*. *I’ve always been an angry person*. *I have serious anger issues especially when I think about how my parents can just abandon me like that as a child*. *If someone does something to me*, *I get very angry and I want us to hit each other”*. ***Interviewer*:** “*Oh*, *and have you ever hit each other*? ***Nomie*: *“****With this one [current partner]*, *no it hasn’t happened*. *I’ve noticed that he holds back even when I try to provoke him so that we can fight he holds back a lot*. *So*, *I tend to push him so that he can get to the point where he hits me so that I can also hit him back*. *But he holds back and doesn’t hit me*. *[laughs]*. *But with the others*? *like my ex*. *We used to fight a lot*. *When we would start fighting everything would be all over the place*, *you’d see this table turned upside down*. *Ah we would beat each other*.*”*

### Experiences of women living with HIV-IPV-MH syndemic

In our study, the women among the group who had experienced all three intersecting epidemics, spontaneously shared their experiences of childhood sexual abuse (CSA), which appears to have contributed to MH challenges in adulthood especially in situations where they disclosed to caregivers and were not believed. It must be noted however, that the lack of disclosure in the other groups, does not preclude the existence of CSA in the histories of the other women who did not disclose. Although we did ask participants about experiences of violence during childhood, we did not explicitly ask about sexual abuse. This could mean that some participants may have chosen not to disclose such experiences. Nonetheless we did find that the MH challenges amongst this group were very severe. Lerato (24), reported:

*“When I was living with my uncle and aunt it was not good*. *They used to abuse me*. *My uncle used to force me to have sex with him*. *He was abusing me*. *This river is where I used to go to be alone and wish that I could die*. *And in terms of the railway*, *I tried to tie myself with a rope and kill myself on the railway*. *But I didn’t manage*. *And even now there are times when I still feel like this*. *Recently I even tried to kill myself*.*”*

When discussing her adult relationships, Lerato shared how she was in an abusive relationship with the father of her child:

*“Ah that one used to hit me a lot*. *He would come home drunk and he would beat me*. *He didn’t do anything for me and my son*. *I had to do everything for us”* **Interviewer:** "*Is there anyone that you told about what was happening*?*”* Lerato: “*No*, *I didn’t tell anyone*. *I’m not used to telling people about my problems because I generally don’t trust people*.”

Sphe (28), who also had an experience of CSA, shared how the experience left her confused and experiencing severe MH challenges, she shared:

*“This is when I was raped [pointing at drawing]*, *I lived with the effects of the rape even though I didn’t know or understand what rape was*. *My family kept telling me that I hadn’t experienced what I knew I went through and that really traumatised me*. *I even started experiencing memory loss and when that happened it was like it validated them and they started saying I am crazy that’s why I was accusing that boy of raping me*. *My life after that was not okay*, *I was constantly scared and paranoid*.*”*

Because of this experience Sphe was isolated from her family, which she believed made her susceptible to dating an abusive partner. When speaking about her partner Sphe recalled:

*“Things between me and my partner started changing and I think they changed because he started learning more about my life and he found out that I am basically an orphan and I have nobody and I rely mostly on him*. *And that’s when he became toxic”* ***Interviewer*:** *“Can you tell me a little bit more about what you mean when you say that he was toxic*? ***Sphe*:** *“One day*, *he asked me why he has never seen anyone in my family coming to visit me in all the time that we had been dating and that’s when I told him about my family background*. *I told him that I do have family but we aren’t close and we don’t visit each other*. *From there on he started changing as he realised that I am alone and that’s when he became toxic and abusive*.*”*

### IPV-HIV syndemic

In relation to the IPV-HIV syndemic, although there was no direct link that didn’t include MH challenges, we did find participants who made their own association, participants shared that they believed that they had contracted HIV because of their abusive partners who had been cheating on them incessantly, Silindile (38) expressed:

*“You know*, *what hurt me the most when I first found out was because I felt like I didn’t deserve to have this thing [HIV]*. *Two sexual partners in my whole life*. *The reality of this really hurt me… I was loyal to my partner*. *I mean you stay with someone and you tell yourself that you are committed but on the other hand you are with a partner who is running the streets*. *And then I discovered I was HIV positive*.*”*

## Coping strategies

Participants from the different groups had varied ways of coping with living with the syndemic and other challenges in their lives. Some of the coping strategies included their own resilience and having the inner belief that they could overcome any obstacle. Religion and church also played a crucial role.

### HIV-MH syndemic and coping strategies

Women living with the HIV-MH syndemic shared that a combination of self-reliance, family support, hope, and religious faith was how they coped. In terms of self-reliance, Rejoice (25) highlighted the significance of having to rely on herself to cope and overcome her adversities:

“*I guess the support system was mine*. *Yes*, *I had uncles and everything*, *but I believe that Rejoice helped herself out of this*, *even though somehow that hurt me*, *but it is also what kept me together to carry on*.*”*

Furthermore, hope, coupled with a solid support system, was emphasized as a crucial component for coping by other participants. Xoli (23), who was born with HIV, and struggled with MH symptoms linked to depression spoke about her hope as well as the support she received from her family in helping her survive to this point:

*“Having the hope that things will get better and being able to confide in my aunt and having her support*… *I have learned that I can overcome anything no matter how difficult it is*.*”*

For some of the women, religious faith and their trust in a higher power were instrumental in navigating their challenges. Their conviction provided both comfort and resilience amid adversity. Nelly (57), who not only contracted HIV from her husband but also grappled with the loss of a child and socio-economic challenges stemming from unemployment, leaned on her faith for comfort She expressed:

*“In everything that I do*, *I say*, *God*, *you are the alpha and omega in my life*, *you will not forsake me*. *I know in the end you will make my life better*.*”*

### IPV-MH syndemic and coping strategies

For women struggling with the IPV-MH syndemic, two primary coping strategies emerged: the use of substances as described earlier for women living with the HIV-IPV-MH syndemic as a means of escape and distraction and clinging to the hope of a brighter future. Londiwe (49), who suffered severe IPV at the hands of her husband, shared that she coped by being hopeful that things would change, she reflected,

*“It was the hope that everything would eventually be fine*. *I hoped that one day things would turn out okay*.*”*

On the subject of substance use as way for mental detachment, Nomie (28) noted:

*“I just drink at home; I drink and then I sleep*. *I usually drink if I want to distract myself and I just don’t want to think*. ***Interviewer*:** *“What do you want to distract yourself from*?*”* ***Nomie*:** *“Honestly*, *everything hey*, *relationships*, *life*, *work*, *everything*. *I sometimes feel like nothing is going right in my life*. *I try do this and it doesn’t work out*, *then I try something else and it also doesn’t work*, *so I find myself feeling discouraged*. *Usually if I don’t drink*, *I’ll end up smoking dagga*. *But I like dagga because when I smoke*, *I don’t think about anything; I just sleep*.*”*

### IPV-MH-HIV syndemic and coping strategies

The women living with the IPV-MH-HIV syndemic, when probed on how they coped with these intersecting epidemics, shared that acceptance, an unwavering commitment to their children, and, for some, substance misuse as a means of temporary relief. Many participants conveyed that alcohol, albeit briefly, provided some form of escape from their lives. Lerato’s (24) narrative paints a vivid picture of her struggle with her HIV diagnosis and her journey towards acceptance. She shared:

*“I’ve accepted it because at the end of the day there is nothing*, *I can do about it”*.

When we probed deeper into the length of her acceptance journey, she touched upon the solace she sought in alcohol not only in relation to her diagnosis but also her childhood trauma experiences:

*“It took a very long time*. *And during that time all I wanted to do was be at the tavern and drink because I didn’t want to think about what had happened*. *I wanted to distract myself*. *I would just go to the tavern*, *drink and come back and pass out*. *If I passed out after a drunk night it was better because I wouldn’t think about anything*.*”*

Diana (43) also shared her story, not only about living with HIV but also grappling with a childhood marred by abuse. The relative who fathered her first child had sexually assaulted her during her younger years. Further complicating her life was the consistent physical and emotional abuse she endured from her husband and the longstanding MH challenges that plagued her from childhood. In search of relief, Diana turned to alcohol. She recounted:

*“He would hit me when he was drunk*… *Then I came back home and decided to never go back there*. *That was the time that I started drinking alcohol*, *I was too stressed*. *I gave my life away to alcohol*, *it felt as though what I was doing was the right thing*. *I felt good when drinking*. *I drank so much to a point that had I not stopped drinking*, *I would have been dead by now”*.

However, amid these challenges, the women’s commitment to motherhood provided a sense of hope for them. Silindile highlighted this sentiment, asserting:

*“I think it’s my children that give me the go forward*, *because the last thing I want is for my children to struggle*.*”* Nokuthula (26) also shared the following when asked how she coped with her challenges, *“My children are the reason that makes me wake up every morning*, *raise my head*, *and say thank God for giving me another day to live*. *Then in my life*, *the people that I have as my priority*, *are my children and my brother”*.

Overall, we found that acceptance was a cross-cutting coping strategy observed across all the different epidemic combinations. Nokuthula (26), who had experienced all three epidemics had the following to say:

*“After being raped*, *the only thing I was scared of was a man*. *I did not want a man near me; they disgusted me*. *So*, *after getting raped for a second time*, *they ran tests on me*, *and that’s where I found out that I have HIV*. *It became difficult for me to accept and live with it*. *It was difficult*, *very difficult*. *But as time went by*, *I decided and accepted that I am living with this thing for the rest of my life and to accept what that man did to me*, *so why should I be miserable because of it*? *I think I have to live with the fact that I am HIV positive and I contracted this through rape*, *and I won’t change it*.”

Moreover, Xoli (23) who was living with the HIV-MH syndemic, shared how she had to accept her diagnosis,

*“It has really affected my family because my younger sister and I were born with HIV*. *At first it was difficult to accept*, *but eventually I accepted that I have to take my medication and go to the clinic in order to live longer*.*”*

Finally, Cindy (49), who was living with the IPV-MH syndemic, shared that when she was being abused by her husband, what helped her cope was hope that he would change as well as acceptance of the situation because of her dependence on him, she reported:

*“I hoped that one day things would turn out okay*. *I was afraid to go to social workers or the police because I didn’t want him to get arrested because if he got arrested what would we eat*. *In life you have to accept everything that happens to you*. *You have to accept and let it pass and understand that this is life*.*”*

Our findings showed that substance use was more commonly mentioned in the combinations involving IPV, whilst hope, self-reliance, family support, and religious faith were prominent coping strategies in the HIV-MH syndemic.

## Discussion

The experiences of women living with the HIV, IPV, and MH syndemic are complex. The study identified intersections and the synergistic interactions between HIV-MH, IPV-MH, and the relationship between IPV-HIV-MH. Our findings suggest that HIV, IPV and MH challenges interact in ways that exacerbate the severity and impact of each condition and we therefore propose that the co-occurrence of these epidemics constitutes a syndemic. Notably, beyond substance use, other MH challenges were evident among the participants, warranting an expansion of the SAVA syndemic concept. We found that among participants living with the HIV- MH syndemic, an HIV diagnosis was met with denial, symptoms related to depression, anxiety and suicidality and delayed treatment initiation. In the IPV-MH syndemic group, participants reported symptoms related to anxiety and depression in relation to the abuse they experienced, participants also reported a bi-directional relationship between IPV and MH with IPV worsening MH and MH challenges increasing women’s vulnerability to IPV. Participants experiencing all three epidemics reported a history of CSA, which was associated with MH challenges over their life course and experiences with IPV from past and current partners. Participants coped with their circumstances mostly through acceptance of the situation and religion, while others used excessive substance use to help them cope and detach. In the groups where IPV was not present, participants relied on family, friends, religion and other social support.

### The experiences of women living with the HIV-MH syndemic

In the HIV-MH interaction, the syndemic nature is evident. We found that an HIV positive diagnosis often triggered MH symptoms linked to depression and anxiety among participants and in many cases also exacerbated the symptoms. This was particularly apparent especially in cases of vertical HIV transmission where maternal guilt, which was often intensified by internalized stigma significantly increased the risk of severe MH challenges such as suicidal ideation. This complex interplay aligns with findings from KwaZulu Natal study which identified a significant prevalence of suicidal ideation among pregnant women diagnosed with HIV [49]. Additionally research from Mpumalanga reported that the factors associated with suicidal ideation among HIV positive pregnant women included, major depression, partner disclosure status, stigma, age, and partner aggression [49–51]. Despite the existing research on HIV positive pregnant women and suicidal ideation, our study identifies a gap in the literature specifically related to maternal suicidal ideation in the context of vertical transmission.

Furthermore, the current study is unique in that it emphasizes the role of internalized stigma within the syndemic. Our findings show that internalized stigma not only amplifies the maternal guilt often associated with vertical transmission but it also intensifies overall MH challenges faced by people living with HIV. Evidently despite the advancements in HIV treatment as well as widespread prevalence, HIV stigma still persists. This finding is significant as it highlights the importance of tackling the medical aspects of HIV, as well as the societal and psychological barriers such as stigma, which significantly contribute to these complex intertwined epidemics.

Moreover, many participants in this group reported initial feelings of overwhelming fear of death and denialism, which led to delays in seeking HIV treatment. Evidently the psychological impact of an HIV diagnosis exacerbated their MH challenges, which in turn impeded them from seeking timely medical intervention. This is in line with other research which has found a positive relationship between poor MH and delayed medical intervention [52]. Such findings highlight the need for psychological screenings alongside HIV testing. Incorporating psychological screenings alongside HIV testing presents multiple advantages. Firstly, it could enable early identification and management of potential MH challenges and as result possibly averting the worsening of both mental and physical outcomes. Secondly, the screening could also serve to address the psychological barriers such as anxiety, fear and denialism that often hinder timely HIV treatment [52]. By implementing psychological screening as part of HIV testing, this can mitigate the syndemic impact of these health challenges.

### The experiences of women living with the IPV- MH syndemic

Women experiencing the IPV-MH syndemic often displayed symptoms indicative of anxiety and depression. For some participants these MH challenges existed before their IPV experience. This aligns with prior research indicating that pre-existing MH challenges can predispose individuals to IPV victimization [11, 53, 54]. Conversely for other participants IPV was a salient trigger or exacerbated MH challenges, creating a cyclical relationship where IPV and MH challenges intensify each other. This bidirectional relationship where each condition aggravates the other is indicative of a syndemic. The MH repercussions of IPV have been thoroughly reported on in the literature, with conditions ranging from PTSD and depression to anxiety and eating disorders [53, 55, 56]. Evidently the relationship between IPV and MH goes beyond co-occurrence. When these two epidemics intersect, they also mutually exacerbate the other, often resulting in a compound impact on the women.

### The HIV-IPV intersection

In the present study, we didn’t find an exclusive overlap between IPV and HIV, as all participants reported concurrent MH challenges. It appeared that any existing IPV-HIV connection was invariably connected with MH challenges. Research indicates that an HIV diagnosis can increase stress at both the individual and relationship level. This heightened stress can adversely impact women’s mental health, potentially increasing their vulnerability to becoming involved in an abusive relationship [9, 57]. Other studies indicate that the influence of HIV on IPV is notably accentuated in women already struggling with IPV in their relationships, as their partners, often with a heightened antisocial disposition, might react more aggressively to an HIV diagnosis disclosure [12].

While we did not find evidence of a standalone IPV-HIV overlap, the data consistently highlighted the omnipresence of MH challenges, intersecting with the other two epidemics. A deeper look at the experiences of women living with the IPV-HIV-MH syndemic is critical at this point.

### The experiences of women living with IPV-HIV-MH syndemic

Among this group of women with the IPV-HIV-MH syndemic, a prominent shared experience was the history of CSA. The trauma from CSA has been identified as a potential precursor to adult MH challenges, given its long-lasting detrimental effects on psychological well-being [58–60]. Many of the participants reported symptoms indicative of depression, anxiety, and suicidality, which align with previous findings on the profound impact of CSA on survivors’ psychosocial development [58, 61].

Furthermore, research indicates that CSA survivors face an elevated risk of sexual revictimization in later life, including within stable relationships [62]. This revictimization can manifest as various forms of IPV, such as psychological and physical abuse [59]. The latter indicates a cyclical pattern where early trauma can lead to MH challenges which in turn increase vulnerability to IPV [54].

Regarding the HIV component, while none of the participants directly linked their HIV diagnosis to violence, some believed that their acquisition resulted from their abusive partners’ extramarital affairs. This perspective underscores the intersectionality of these epidemics: the compounded vulnerabilities from CSA and MH challenges potentially leading to relationships characterised by IPV, and within these relationships, an increased risk of contracting HIV.

It must be noted, however, that these relationships are not strictly linear but bidirectional. For instance, while CSA can predispose individuals to MH challenges [61], which might then lead to violent relationships and an elevated HIV risk [12, 59], the reverse can also hold true. MH challenges might manifest first, making the child more susceptible to CSA. Similarly, an HIV diagnosis might precipitate or exacerbate violence in relationships [12]. Recognizing this intricate web of bidirectional influences underscores the need for a nuanced and multifaceted approach to understanding and addressing the overlapping epidemics faced by these women.

## Coping strategies

According to Lazarus and Folkman (1984) two primary coping modalities can exist: problem-focused and emotion-focused coping [63]. The former encompasses efforts to alter the environment or the problem itself, while the latter aims to alleviate emotional distress [63]. Most of our participants leaned towards emotion-focused coping strategies. The coping strategies used by women living with different combinations of the IPV, HIV and MH syndemic revealed several patterns and similarities. Acceptance emerged as a cross-cutting coping strategy observed across all combinations. Participants in the HIV-MH group expressed religion and belief in God as important in coping with challenges. Additionally, substance use was commonly mentioned in combinations involving IPV. Furthermore, the reliance of social support to cope was found in the groups who did not experience IPV. Research focusing on coping strategies among women dealing with IPV revealed that victims found their families, friends, and neighbours to be supportive and reliable resources for assistance [25]. Our study, however, did not find that participants experiencing IPV relied on family and social support structures as a way of coping, in such situations. Some theories have posited that the reason for this could be that often in IPV situations the perpetrator is likely to isolate their partner from their friends and family [64].

The notion of acceptance although cross-cutting across the groups, its manifestation differed based on the unique combination of adversities each participant faced. When considering HIV and acceptance, our findings resonate with prior research in the field. For example, a study by Sreelekshmi (2015) involving 150 HIV-positive individuals highlighted the prevalent use of emotion-focused coping techniques [23]. A significant 65.7% of participants identified acceptance as their primary coping approach. However, for some participants who had experienced IPV, the acceptance conveyed often leaned towards resignation. This type of acceptance was deeply entrenched in feelings of desolation and helplessness. Moreover, in the literature, acceptance in the face of IPV has also been characterised as a form of denial or avoidance which is often linked to PTSD symptomology [65–67]. On the other hand, there were some participants that seemed to have accepted and normalized the violence. In relation to the latter, research has also found that in some contexts young women may perceive violent men as more desirable partners and as being real men, in such situations, IPV is not only normalized but is also seen as a necessary component in romantic relationship [68, 69].

As mentioned, substance misuse, especially in the context of IPV was a recurrent theme amongst participants. This observation is echoed by Mehr et al. (2023), who define substance misuse as the consumption of substances in high quantities or inappropriate contexts [70]. Their research highlights that those subjected to IPV frequently resort to substance misuse as a way to manage the emotional and physical trauma they endure and as a form of self-medication. Our findings are in line with this, particularly among women who had experienced the combinations including IPV. Our findings add and expand to the literature on coping strategies among women living with the different combinations of HIV, IPV and MH epidemics, as we not only identified which strategies were more prevalent but also how their manifestations differed based on the unique combinations of epidemics each woman faced.

Understanding how women cope with these often-co-occurring epidemics is imperative as it provides an opportunity to better inform clinical practice, as well as contribute to the development of comprehensive, evidence-based strategies for prevention, support, and treatment. Furthermore, our findings also highlight the significance of considering the impact at the community and interpersonal levels in IPV research. They also affirm the perspective that IPV should be treated as a communal issue rather than an individual problem.

## Limitations

Although the current study provides valuable insights, it is not without its shortcomings and limitations. The first limitation is related to translation of the interviews. Some researchers argue that translation during the research process has the potential to introduce a level of bias as translation involves some subjective interpretation [71]. It includes cultural interpretation and an understanding of the context, and herein is where the bias is often introduced [72]. It is thus critical to incorporate ethical translation practices in research to limit such biases. These include using translators who are not only proficient in the language but are also familiar with the context and the environment [73]. For the current research, interviews were conducted in Siswati and Xitsonga and subsequently transcribed and translated into English. Though this posed a potential limitation as some nuances may have been lost in translation, efforts were made to ensure accuracy and preserve the essence of the participants’ responses. The measures included, having transcribers who were fluent in the language the interview was conducted in as well as English. Secondly the interviewers were also familiar with the community, with two of the interviewers having worked in the area for over a decade on various other research projects. Finally, in cases where there was no direct translation for a particular word or phrase, an explanation in English was provided in brackets.

Another limitation concerns the method used for categorizing participants into different epidemic combinations. While this approach initially provided a structured framework for analysis, it might have introduced some degree of arbitrariness, where in some cases some participants may not have met the criteria of the questionnaire leading them to not being categorized as experiencing a particular MH challenge. However, to refine this categorization, we re-categorized the groups, based on the information gathered from the qualitative interviews. For example, participants not initially flagged for MH challenges in the questionnaire but who later revealed symptoms indicative of such during the interviews were re-categorized accordingly. This additional layer of categorization aimed to capture more accurately the complexities of each participant’s experiences.

Although there is much debate about what constitutes as sensitive research, according to Lee and Renzetti, any topic can be deemed as sensitive, however there are some topics that are likely to cause more distress in individuals than others such as HIV/AIDS, MH issues, death and bereavement [74, 75]. Research on personal experiences of violence and childhood trauma can be classified as sensitive [76]. The sensitive nature of the topics discussed in the interviews may have influenced the participant’s willingness to fully share and be open, thus leading to potential underreporting due to discomfort or fear of stigma. To address this, interviewers assured participants of the confidentiality of their responses, and assurance that they would receive help if they felt distressed during the interview. Ensuring participants anonymity and confidentiality increased the likelihood of participants providing more truthful and genuine responses [77].

Reflexivity in qualitative research is imperative, this involves the ongoing, cooperative and multifaceted activities where researchers actively critique and assess the ways in which their personal perspectives and backgrounds can potentially affect the research [78]. We acknowledge that our personal beliefs, cultural understanding, and personal experiences may have shaped our interpretation as well as the presentation of the participants’ stories and as a result potentially introducing subjective biases. As an attempt to mitigate this, the research team had regular debriefing sessions throughout the study, where we discussed and challenged each other’s viewpoints, which helped in ensuring a more balanced perspective on the data. Furthermore, the interviewers also kept detailed field notes after the interviews which were reflective accounts of the interviews including the interviewer thoughts and reactions to the interview.

Furthermore, the potential for response bias due to the interview-led nature of the interviews was another limitation. However, to mitigate this, experienced qualitative interviewers were used and trained to maintain neutrality and foster a non-judgmental and supportive environment.

Finally, while the study’s focus on the South African peri-urban and rural context provides in-depth and context specific insights especially due to the limited data in such settings, this also implies that the findings may not be directly transferable to other settings. The experience of women in this specific context are shaped by unique socio-cultural, economic, and environmental factors which may differ significantly from those in other provinces or countries.

Despite the limitations outlined, the study holds substantial value, particularly in understanding the experiences of women in South African peri-urban and rural contexts. It also lays a foundation for further research, suggesting the need for more studies with diverse samples to broaden the understanding of these complex experiences in varied settings.

## Implications of the research and next steps

The findings of the current study highlight the importance of addressing the interconnected epidemics of HIV, IPV and MH in a comprehensive and integrated manner. A key implication is the need for holistic care approaches that are geared towards tackling these interlinked epidemics rather than treating them in isolation. A holistic approach has the potential to improve prevention and response strategies, ultimately contributing to beneficial outcomes in public health [79]. A good example of this is the SHARE multicomponent intervention in Uganda which has effectively managed to reduce HIV incidence and IPV in women [80].

Furthermore, taking into consideration the diversity in coping strategies observed in the participants, there is a need to support positive coping strategies while providing targeted interventions for harmful coping strategies such as substance misuse among women living with the syndemic. An example is the coping strategies intervention implemented at the ART clinic of the All-India Institute of Medical Science in New Delhi. The intervention aimed to enhance positive coping mechanisms and quality of life among people living with HIV through informational support, adaptive coping strategies, social support, and positive living [81].

Furthermore, our study highlights the importance of considering the unique contexts and backgrounds of individuals when designing and implementing public health interventions. In particular, the role of cultural and socio-economic factors in shaping the experiences of HIV, IPV and MH epidemics requires context-specific strategies.

Moreover, the high prevalence of MH challenges identified in the current sample highlights a need for enhancement of MH care especially in peri-urban and rural areas, where MH services are close to non-existent [70]. Strengthening MH services in these areas would serve as a critical aspect in addressing the syndemic effectively. Research highlights the importance of community-based MH interventions as pivotal especially in low-resource communities. For instance, Malawi has integrated their Health Surveillance Assistants to deliver MH interventions into the community’s already existing social and cultural structures [82]. Similarly, in South Africa there is an ongoing effort through the Mental Health integration program aimed at enhancing integration of MH services in the primary health care system. However, despite the innovativeness of this program, the effectiveness is significantly hindered by inadequate funding, poor administrative coordination and low MH awareness among officials and the general population. [83].

Future research should focus on the development of interventions that can address the co-occurrence and the multi-faceted nature of these epidemics. This includes the exploration of the efficacy of integrated care models and the potential of Mobile-health solutions in increasing accessibility to MH and support services, especially in resource limited settings. There is also a need for more qualitative research on the IPV, MH and HIV syndemic. Whilst there is an abundance of quantitative research in this area [84–87], qualitative research is critical in providing a deeper and more nuanced understanding of the syndemic.

## Conclusion

Our study highlights the intricate hardships faced by women navigating combinations of HIV, IPV, and MH epidemics and how the combination of epidemics worsens each of these. MH challenges were a consistent factor, whether associated with HIV, or IPV. A deeper understanding into these women’s multifaceted experiences is vital for developing targeted strategies that address their specific needs, thus enhancing their overall quality of life.

## Supporting information

S1 Table Referral flags and categories. (DOCX)

S1 Text Interview guide. (DOCX)

S2 Text Art-based method prompts. (DOCX)

S1 Checklist STROBE statement—Checklist of items that should be included in reports of observational studies. (DOCX)

S2 Checklist Inclusivity in global research. (DOCX)

## References

[1] Brandt R. The mental health of people living with HIV/AIDS in Africa: a systematic review. African Journal of AIDS Research. 2009 Jun;8(2):123–33. doi: 10.2989/AJAR.2009.8.2.1.853 25875564

[2] Remien RH, Stirratt MJ, Nguyen N, Robbins RN, Pala AN, Mellins CA. Mental health and HIV/AIDS: the need for an integrated response. AIDS. 2019 Jul 15;33(9):1411–20. doi: 10.1097/QAD.0000000000002227 30950883 PMC6635049

[3] Oram S, Fisher HL, Minnis H, Seedat S, Walby S, Hegarty K, et al. The Lancet Psychiatry Commission on intimate partner violence and mental health: advancing mental health services, research, and policy. The Lancet Psychiatry. 2022 Jun;9(6):487–524. doi: 10.1016/S2215-0366(22)00008-6 35569504

[4] Noble RE. Depression in women. Metabolism. 2005 May;54(5):49–52. doi: 10.1016/j.metabol.2005.01.014 15877314

[5] McLean CP, Anderson ER. Brave men and timid women? A review of the gender differences in fear and anxiety. Clinical Psychology Review. 2009 Aug;29(6):496–505. doi: 10.1016/j.cpr.2009.05.003 19541399

[6] Breslau N, Anthony JC. Gender differences in the sensitivity to posttraumatic stress disorder: An epidemiological study of urban young adults. Journal of Abnormal Psychology. 2007 Aug;116(3):607–11. doi: 10.1037/0021-843X.116.3.607 17696716

[7] Breslau N. Sex Differences in Posttraumatic Stress Disorder. Arch Gen Psychiatry. 1997 Nov 1;54(11):1044. doi: 10.1001/archpsyc.1997.01830230082012 9366662

[8] Orza L, Bewley S, Logie CH, Crone ET, Moroz S, Strachan S, et al. How does living with HIV impact on women’s mental health? Voices from a global survey. J Int AIDS Soc. 2015;18(Suppl 5):20289. doi: 10.7448/IAS.18.6.20289 26643460 PMC4672402

[9] Devries KM, Mak JY, Bacchus LJ, Child JC, Falder G, Petzold M, et al. Intimate Partner Violence and Incident Depressive Symptoms and Suicide Attempts: A Systematic Review of Longitudinal Studies. Tsai AC, editor. PLoS Med. 2013 May 7;10(5):e1001439. doi: 10.1371/journal.pmed.1001439 23671407 PMC3646718

[10] Seedat S. Interventions to Improve Psychological Functioning and Health Outcomes of HIV-Infected Individuals with a History of Trauma or PTSD. Curr HIV/AIDS Rep. 2012 Dec;9(4):344–50. doi: 10.1007/s11904-012-0139-3 23007792

[11] Woollett N, Hatcher AM. Mental health, intimate partner violence and HIV. S Afr Med J. 2016 Sep 10;106(10):969.

[12] Sulivan TP. The Intersection of Intimate Partner Violence and HIV: Detection, Disclosure, Discussion, and Implications for Treatment Adherence. Topics in Antiviral Medicine. 2019 May;27(2):84–7. 31136996 PMC6550354

[13] Singer M. A dose of drugs, a touch of violence, a case of AIDS: Conceptualizing the SAVA syndemic. Free Inquiry—Special issue: Gangs, Drugs and Violence. 1996;24(2).

[14] World Health Organization. Epidemiological Fact Sheet: HIV Statistics, globally and by WHO region, 2023. 2023.

[15] UNAIDS. Epidemiological Estimates. UNAIDS; 2023. unaids.org/sites/default/files/media_asset/data-book-2023_en.pdf

[16] Norman R, Schneider M, Bradshaw D, Jewkes R, Abrahams N, Matzopoulos R, et al. Interpersonal violence: an important risk factor for disease and injury in South Africa. Popul Health Metrics. 2010 Dec;8(1):32. doi: 10.1186/1478-7954-8-32 21118578 PMC3009696

[17] World Health Organization. Mental health [Internet]. 2023. https://www.who.int/health-topics/mental-health#tab=tab_2

[18] Krause ED, Kaltman S, Goodman LA, Dutton MA. Avoidant coping and PTSD symptoms related to domestic violence exposure: A longitudinal study. Journal of Traumatic Stress. 2008 Feb;21(1):83–90. doi: 10.1002/jts.20288 18302182

[19] Goodman L, Dutton MA, Weinfurt K, Cook S. The Intimate Partner Violence Strategies Index: Development and Application. Violence Against Women. 2003 Feb;9(2):163–86.

[20] Brandão BMGDM, Angelim RCDM, Marques SC, Oliveira RCD, Abrão FMDS. Living with HIV: coping strategies of seropositive older adults. Rev esc enferm USP. 2020;54:e03576. doi: 10.1590/s1980-220x2018027603576 32667387

[21] Burgess R, Campbell C. Contextualising women’s mental distress and coping strategies in the time of AIDS: A rural South African case study. Transcult Psychiatry. 2014 Dec;51(6):875–903. doi: 10.1177/1363461514526925 24670517

[22] Medley AM, Kennedy CE, Lunyolo S, Sweat MD. Disclosure Outcomes, Coping Strategies, and Life Changes Among Women Living With HIV in Uganda. Qual Health Res. 2009 Dec;19(12):1744–54. doi: 10.1177/1049732309353417 19949223

[23] Sreelekshmi R. Anxiety and coping mechanisms among HIV positive patients. Manipal Journal of Nursing and Health Sciences. 2015 Jul;1(2):6.

[24] Rizo CF, Givens A, Lombardi B. A systematic review of coping among heterosexual female IPV survivors in the United States with a focus on the conceptualization and measurement of coping. Aggression and Violent Behavior. 2017 May;34:35–50.

[25] Sere Y, Roman NV, Ruiter RAC. Coping With the Experiences of Intimate Partner Violence Among South African Women: Systematic Review and Meta-Synthesis. Front Psychiatry. 2021;12:655130. doi: 10.3389/fpsyt.2021.655130 34122178 PMC8187566

[26] Bleakley A. Stories as data, data as stories: making sense of narrative inquiry in clinical education: original article. Medical Education. 2005 May;39(5):534–40. 15842721 10.1111/j.1365-2929.2005.02126.x

[27] Lieblich A, Tuval-Mashiach R, Zilber T. Narrative research: reading, analysis and interpretation. Thousand Oaks, Calif: Sage Publications; 1998. 185 p. (Applied social research methods series).

[28] Hoshmand LT. Narratology, cultural psychology, and counseling research. Journal of Counseling Psychology. 2005 Apr;52(2):178–86.

[29] Simbayi LC, Zuma K, Zungu N, Moyo S, Marinda E, Jooste S, et al. South African National HIV Prevalence, Incidence, Behavior and Communication Survey, 2017. Cape Town: HSRC Press; 2017. hsrc.ac.za/uploads/pageContent/10779/SABSSM%20V.pdf

[30] South African High Commission (Ottawa). South Africa’s Nine Provinces [Internet]. South Africa’s Nine Provinces. 2019 [cited 2023 Aug 7]. http://www.southafrica-canada.ca/south-africas-nine-provinces/#:~:text=Mpumalanga%3A,%25)%20and%20isiNdebele%20(10%25).

[31] Gómez-Olivé FX, Angotti N, Houle B, Klipstein-Grobusch K, Kabudula C, Menken J, et al. Prevalence of HIV among those 15 and older in rural South Africa. AIDS Care. 2013 Sep;25(9):1122–8. doi: 10.1080/09540121.2012.750710 23311396 PMC3778517

[32] Seabi A Tshidi. Marriage, Cohabitation and Domestic Violence in Mpumalanga. University of Pretoria; 2009. repository.up.ac.za/bitstream/handle/2263/27841/dissertation.pdf?sequence=1&isAllowed=y

[33] Herman AA, Stein DJ, Seedat S, Heeringa SG, Moomal H, Williams DR. The South African Stress and Health (SASH) study: 12-month and lifetime prevalence of common mental disorders. S Afr Med J. 2009 May;99(5 Pt 2):339–44. 19588796 PMC3191537

[34] Meinck F, Woollett N, Franchino-Olsen H, Silima M, Thurston C, Fouché A, et al. Interrupting the intergenerational cycle of violence: protocol for a three-generational longitudinal mixed-methods study in South Africa (INTERRUPT_VIOLENCE). BMC Public Health. 2023 Feb 27;23(1):395. doi: 10.1186/s12889-023-15168-y 36849941 PMC9969039

[35] Horowitz LM, Bridge JA, Teach SJ, Ballard E, Klima J, Rosenstein DL, et al. Ask Suicide-Screening Questions (ASQ): A Brief Instrument for the Pediatric Emergency Department. Arch Pediatr Adolesc Med. 2012 Dec 1;166(12):1170. doi: 10.1001/archpediatrics.2012.1276 23027429 PMC6889955

[36] Löwe B, Decker O, Müller S, Brähler E, Schellberg D, Herzog W, et al. Validation and Standardization of the Generalized Anxiety Disorder Screener (GAD-7) in the General Population. Medical Care. 2008 Mar;46(3):266–74. doi: 10.1097/MLR.0b013e318160d093 18388841

[37] Adewuya AO, Ola BA, Afolabi OO. Validity of the patient health questionnaire (PHQ-9) as a screening tool for depression amongst Nigerian university students. Journal of Affective Disorders. 2006 Nov;96(1–2):89–93. doi: 10.1016/j.jad.2006.05.021 16857265

[38] Hansen M, Andersen TE, Armour C, Elklit A, Palic S, Mackrill T. PTSD-8: A Short PTSD Inventory. CPEMH. 2010 Sep 28;6(1):101–8. 10.2174/1745017901006010101 PMC3023946 21253461

[39] Garcia-Moreno C, Ellsberg M, Heise L. WHO multi-country study study on women’s health and domestic violence against women: summary report: initial results on prevalence, health outcomes and women’s responses. Geneva, Switzerland: World Health Organization; 2005. https://www.who.int/publications/i/item/924159358X

[40] Burns RC, Kaufman SH. Action, Styles, And Symbols In Kinetic Family Drawings Kfd [Internet]. Routledge; 2013 May [cited 2023 May 17]. https://www.taylorfrancis.com/books/9781135064211

[41] Denov M, Shevell MC. An arts-based approach with youth born of genocidal rape in Rwanda: The river of life as an autobiographical mapping tool. Global Studies of Childhood. 2021 Mar;11(1):21–39. doi: 10.1177/2043610621995830 33868636 PMC8022799

[42] Mannay D. Visual, narrative and creative research methods: application, reflection and ethics. 1 Edition. London: Routledge, Taylor & Francis Group; 2016. 152 p.

[43] Ebersöhn L, Eloff I, Finestone M, Department of Educational Psychology, University of Pretoria, Pretoria, South Africa, Van Dullemen I, Sikkema K, et al. Drawing on resilience: piloting the utility of the Kinetic Family Drawing to measure resilience in children of HIV-positive mothers. SAJE. 2012 Nov 7;32(4):331–48.

[44] Weinrib EL. Images of the self: the sandplay therapy process. 2nd ed. Cloverdale, CA: Temenos Press; 2004. 234 p.

[45] Braun V, Clarke V. Thematic analysis: a practical guide. London; Thousand Oaks, California: SAGE; 2022. 338 p.

[46] Creswell JW, Creswell JW. Qualitative inquiry & research design: choosing among five approaches. 2nd ed. Thousand Oaks: Sage Publications; 2007. 395 p.

[47] Kalff DM. Sandplay: a psychotherapeutic approach to the psyche. Santa Monica, CA: Sigo Press; 1980. 169 p.

[48] Lowenfeld M. The Nature and use of the Lowenfeld World Technique in Work with Children and Adults. The Journal of Psychology. 1950 Oct;30(2):325–31.

[49] Rochat TJ, Mkwanazi N, Bland R. Maternal HIV disclosure to HIV-uninfected children in rural South Africa: a pilot study of a family-based intervention. BMC Public Health. 2013 Dec;13(1):147. doi: 10.1186/1471-2458-13-147 23418933 PMC3599138

[50] Rodriguez VJ, Mandell LN, Babayigit S, Manohar RR, Weiss SM, Jones DL. Correlates of Suicidal Ideation During Pregnancy and Postpartum Among Women Living with HIV in Rural South Africa. AIDS Behav. 2018 Oct;22(10):3188–97. doi: 10.1007/s10461-018-2153-y 29752621 PMC6230517

[51] Zegeye A, Alebel A, Gebrie A, Tesfaye B, Belay YA, Adane F, et al. Prevalence and determinants of antenatal depression among pregnant women in Ethiopia: a systematic review and meta-analysis. BMC Pregnancy Childbirth. 2018 Dec;18(1):462. doi: 10.1186/s12884-018-2101-x 30486804 PMC6264030

[52] Rane MS, Hong T, Govere S, Thulare H, Moosa MY, Celum C, et al. Depression and Anxiety as Risk Factors for Delayed Care-Seeking Behavior in Human Immunodeficiency Virus–Infected Individuals in South Africa. Clinical Infectious Diseases. 2018 Oct 15;67(9):1411–8. doi: 10.1093/cid/ciy309 29659757 PMC6186861

[53] Devries KM, Knight L, Child JC, Kyegombe N, Hossain M, Lees S, et al. Witnessing intimate partner violence and child maltreatment in Ugandan children: a cross-sectional survey. BMJ Open. 2017 Feb 28;7(2):e013583. doi: 10.1136/bmjopen-2016-013583 28246136 PMC5337724

[54] Williams DR, Herman A, Stein DJ, Heeringa SG, Jackson PB, Moomal H, et al. Twelve-month mental disorders in South Africa: prevalence, service use and demographic correlates in the population-based South African Stress and Health Study. Psychol Med. 2008 Feb;38(2):211–20. doi: 10.1017/S0033291707001420 17903333 PMC2718686

[55] Coker AL. Toward Violence Intervention and Prevention: Reflections on “Physical and Mental Health Effects of Intimate Partner Violence for Men and Women”. American Journal of Preventive Medicine. 2021 Dec;61(6):771–3. doi: 10.1016/j.amepre.2021.08.001 34801205

[56] Ellsberg M, Emmelin M. Intimate Partner Violence and Mental Health. Global Health Action. 2014 Dec;7(1):25658. doi: 10.3402/gha.v7.25658 25226422 PMC4165041

[57] Hand GA, Phillips KD, Dudgeon WD. Perceived stress in HIV-infected individuals: Physiological and psychological correlates. AIDS Care. 2006 Nov;18(8):1011–7. doi: 10.1080/09540120600568376 17012093

[58] Bulik CM, Prescott CA, Kendler KS. Features of childhood sexual abuse and the development of psychiatric and substance use disorders. Br J Psychiatry. 2001 Nov;179(5):444–9. doi: 10.1192/bjp.179.5.444 11689403

[59] Herbert A, Heron J, Barter C, Szilassy E, Barnes M, Howe LD, et al. Risk factors for intimate partner violence and abuse among adolescents and young adults: findings from a UK population-based cohort. Wellcome Open Res. 2021 Jan 21;5:176. doi: 10.12688/wellcomeopenres.16106.3 33553678 PMC7848855

[60] Scoglio AAJ, Kraus SW, Saczynski J, Jooma S, Molnar BE. Systematic Review of Risk and Protective Factors for Revictimization After Child Sexual Abuse. Trauma, Violence, & Abuse. 2021 Jan;22(1):41–53. doi: 10.1177/1524838018823274 30669947

[61] Fergusson DM, McLeod GFH, Horwood LJ. Childhood sexual abuse and adult developmental outcomes: Findings from a 30-year longitudinal study in New Zealand. Child Abuse & Neglect. 2013 Sep;37(9):664–74. doi: 10.1016/j.chiabu.2013.03.013 23623446

[62] Dube SR, Anda RF, Felitti VJ, Chapman DP, Williamson DF, Giles WH. Childhood Abuse, Household Dysfunction, and the Risk of Attempted Suicide Throughout the Life Span: Findings From the Adverse Childhood Experiences Study. JAMA. 2001 Dec 26;286(24):3089. doi: 10.1001/jama.286.24.3089 11754674

[63] Biggs A, Brough P, Drummond S. Lazarus and Folkman’s Psychological Stress and Coping Theory. In: Cooper CL, Quick JC, editors. The Handbook of Stress and Health [Internet]. Chichester, UK: John Wiley & Sons, Ltd; 2017 [cited 2023 Jun 15]. p. 349–64. https://onlinelibrary.wiley.com/doi/10.1002/9781118993811.ch21

[64] Goodson A, Hayes BE. Help-Seeking Behaviors of Intimate Partner Violence Victims: A Cross-National Analysis in Developing Nations. J Interpers Violence. 2021 May;36(9–10):NP4705–27. doi: 10.1177/0886260518794508 30136887

[65] Chadambuka C. Coping Strategies Adopted by Women Who Experienced Intimate Partner Violence in the Context of Social Norms in Rural Areas in Zimbabwe. J Interpers Violence. 2022 Mar;37(5–6):2776–800. doi: 10.1177/0886260520943734 32697141

[66] Foster EL, Becho J, Burge SK, Talamantes MA, Ferrer RL, Wood RC, et al. Coping with intimate partner violence: Qualitative findings from the study of dynamics of husband to wife abuse. Families, Systems, & Health. 2015 Sep;33(3):285–94. 10.1037/fsh0000130 26053568

[67] Rodriguez TJ. Psychological Well-Being and Coping Mechanisms of Battered Women. AJOH [Internet]. 2011 Jan 25 [cited 2023 Sep 14];1(1). Available from: http://asianscientificjournals.com/new/publication/index.php/ajoh/article/view/158

[68] Zembe YZ, Townsend L, Thorson A, Silberschmidt M, Ekstrom AM. Intimate Partner Violence, Relationship Power Inequity and the Role of Sexual and Social Risk Factors in the Production of Violence among Young Women Who Have Multiple Sexual Partners in a Peri-Urban Setting in South Africa. Braitstein P, editor. PLoS ONE. 2015 Nov 23;10(11):e0139430. doi: 10.1371/journal.pone.0139430 26599394 PMC4658116

[69] Hunt X, Van Der Merwe A, Swartz L, Xakayi W, Chideya Y, Hartmann L, et al. “It is in the Nature of Men”: The Normalization of Non-Consensual Sex and Intimate Partner Violence Against Women with Acquired Physical Disabilities in South Africa. Violence Against Women. 2023 May 2;107780122311727. doi: 10.1177/10778012231172710 37132035 PMC11316339

[70] Mehr JB, Bennett ER, Price JL, De Souza NL, Buckman JF, Wilde EA, et al. Intimate partner violence, substance use, and health comorbidities among women: A narrative review. Front Psychol. 2023 Jan 27;13:1028375. doi: 10.3389/fpsyg.2022.1028375 36778165 PMC9912846

[71] Edwards R. A critical examination of the use of interpreters in the qualitative research process. Journal of Ethnic and Migration Studies. 1998 Jan;24(1):197–208.

[72] Hodges P. The application of Berman’s Theory as a Basis for target text evaluation. The AALITRA Review: A journal of literary tranlsation. 2016;48–59.

[73] Qoyyimah U. Handling translations of data for qualitative research. FLS. 2023 Feb 6;5(1):1.

[74] Lee RM, Renzetti CM. The Problems of Researching Sensitive Topics: An Overview and Introduction. American Behavioral Scientist. 1990 May;33(5):510–28.

[75] Davis M, Bolding G, Hart G, Sherr L, Elford J. Reflecting on the experience of interviewing online: perspectives from the Internet and HIV study in London. AIDS Care. 2004 Nov;16(8):944–52. doi: 10.1080/09540120412331292499 15511726

[76] Fraga S. Methodological and ethical challenges in violence research. Porto Biomedical Journal. 2016 May;1(2):77–80. doi: 10.1016/j.pbj.2016.04.005 32258554 PMC6806949

[77] Kang E, Hwang HJ. The importance of anonymity and confidentiality for conducting survey research. Journal of Research adn Publication Ethics. 2023;4(1):1–7.

[78] Olmos-Vega FM, Stalmeijer RE, Varpio L, Kahlke R. A practical guide to reflexivity in qualitative research: AMEE Guide No. 149. Medical Teacher. 2023 Mar 4;45(3):241–51. 10.1080/0142159X.2022.2057287 35389310

[79] Tsagkarliotis I, Rachaniotis NP. A holistic approach in epidemics. Front Public Health. 2023 Nov 13;11:1263293. doi: 10.3389/fpubh.2023.1263293 38026383 PMC10679669

[80] Wagman JA, Gray RH, Campbell JC, Thoma M, Ndyanabo A, Ssekasanvu J, et al. Effectiveness of an integrated intimate partner violence and HIV prevention intervention in Rakai, Uganda: analysis of an intervention in an existing cluster randomised cohort. The Lancet Global Health. 2015 Jan;3(1):e23–33. doi: 10.1016/S2214-109X(14)70344-4 25539966 PMC4370228

[81] Khakha D, Kapoor B. Effect of coping strategies intervention on the coping styles adopted by People living with HIV/AIDS at a tertiary care hospital in the national capital. Nursing & Midwifery Research Journal. 2015 Apr;11(2):45–56.

[82] Wright J. Strengthening mental health care in Southern Malawi: contested meanings and the search for culturally embedded approaches. University of New York; 2021.

[83] Lovero KL, Lammie SL, Van Zyl A, Paul SN, Ngwepe P, Mootz JJ, et al. Mixed-methods evaluation of mental healthcare integration into tuberculosis and maternal-child healthcare services of four South African districts. BMC Health Serv Res. 2019 Dec;19(1):83. doi: 10.1186/s12913-019-3912-9 30704459 PMC6357439

[84] Sherr L, Skeen S, Hensels IS, Tomlinson M, Macedo A. The effects of caregiver and household HIV on child development: a community-based longitudinal study of young children: Caregiver HIV and child development. Child: Care, Health and Development. 2016 Nov;42(6):890–9. 27514630 10.1111/cch.12387 PMC6086490

[85] Levendosky AA, Huth-Bocks AC, Shapiro DL, Semel MA. The impact of domestic violence on the maternal-child relationship and preschool-age children’s functioning. Journal of Family Psychology. 2003;17(3):275–87. doi: 10.1037/0893-3200.17.3.275 14562453

[86] Murphy DA, Marelich WD, Armistead L, Herbeck DM, Payne DL. Anxiety/stress among mothers living with HIV: effects on parenting skills and child outcomes. AIDS Care. 2010 Dec;22(12):1449–58. doi: 10.1080/09540121.2010.487085 20824552 PMC3000905

[87] Thurston IB, Howell KH, Kaufman CC, Mandell JE, Decker KM. Parenting in matched pairs of women of color experiencing intimate partner violence and living with and without HIV. Journal of Traumatic Stress. 2021 Oct;34(5):1005–15. doi: 10.1002/jts.22737 34637554

