# Supplementary material for: “I’ve accepted it because at the end of the day there is nothing, I can do about it”: A qualitative study exploring the experiences of women living with the HIV, intimate partner violence and mental health syndemic in Mpumalanga, South Africa
Source: PLOS Glob Public Health. 2024 May 6;4(5):e0002588. doi: 10.1371/journal.pgph.0002588 (PMC11073682; doi:10.1371/journal.pgph.0002588)
Supplement: S1 Table — (DOCX) [file pgph.0002588.s001.docx]

**S1 Table: Referral Flags and Categories**

| **Referral Category** | **Action steps for fieldworker** |
| --- | --- |
|  |  |
| CODE Black / Adult suicidality (disclosed or observed) | Discuss with participant that we think they need help with their mental health and the need for referral to Social Worker and health services. If they agree, make urgent referral to Social Worker. If they disagree, make referral to Social Worker, and discuss with her but highlight that they did not want further involvement. |
| CODE Purple / Domestic violence, no children in the home & no weapons (disclosed) | Participant has disclosed domestic violence. Offer support through Social Worker, if participant agrees, make referral to Social Worker; choose correct category based on whether weapons are involved. |
| CODE Purple / Domestic violence, no children in the home & weapons (disclosed) |  |
| CODE Red / Sexual abuse or violence experienced by a current adult (disclosed) | Participant has disclosed sexual violence experience. Offer support through Social Worker, if participant agrees, make referral to Social Worker. If rape happened within the last 72 hours, tell participant they should be seen urgently at a clinic to receive post-exposure prophylaxis and if female pregnancy prophylaxis. IF participant agrees to see Social Worker, make this an urgent referral. |
| CODE Navy / Psychosis or mental health issues (disclosure) | Discuss with participant that you think it would be good for them to be referred to mental health services and suggest they talk to Social Worker. If they agree, make referral to Social Worker. |
| CODE H / Positive HIV test | participant has tested positive to HIV. Refer to the clinic for follow-up. Refer to Social Worker if they have any biological child/children to ensure they are followed up and HIV testing for child is arranged. |
