# Supplementary material for: “I’ve accepted it because at the end of the day there is nothing, I can do about it”: A qualitative study exploring the experiences of women living with the HIV, intimate partner violence and mental health syndemic in Mpumalanga, South Africa
Source: PLOS Glob Public Health. 2024 May 6;4(5):e0002588. doi: 10.1371/journal.pgph.0002588 (PMC11073682; doi:10.1371/journal.pgph.0002588)
Supplement: S1 Text — (DOCX) [file pgph.0002588.s002.docx]

**Supplementary File 2: Interview Guide**

In-depth interview guide – young adults

NOTE: This interview will be recorded, ensure consent given for recording

INTRODUCTION

Thank you for agreeing to talk with me today. I am interested in relationships between people and your opinion matters to me.

The questions I have don’t have any right or wrong answers. I am interested in any experiences, stories, and ideas you’d like to share. Please feel free to share your honest thoughts and opinions. Remember that what is said here today is confidential (which means that I can’t talk to others about what we talk about unless you tell me about a child in danger. If that is the case I will have to alert our social worker). When I share our conversation today for research purposes, I will never use your name or anything that can identify you.

We will talk for about 45 minutes and will also draw. We can take a break whenever you would like. Do you have any questions for me before we begin?

Great. Let’s get started.

INTERVIEW GUIDE:

Violence:

1. Please tell me about any challenges or difficult times you’ve experienced?

Ngicela ungitjele ngetigcinamba loke wabukana nato nobe bumatima loke wandlula kubo?

1. Tell me about any times you were hurt by anyone including a family member or partner or someone else? Who did you tell and what happened? *(NOTE: probe in detail about the event)* Ngicela ungitjele ngetikhatsi lapho uke walinyatwa nobe wahlukubetwa khona ngumuntfu, kungaba nalilunge lemndeni, u mlingani wakho nanobe ngubani nje? Watjela bani futsi kwentekani?
2. Tell me about other times when you have been hurt in your life? (NOTE: repeat question multiple times)kukhona yini letinye tikhatsi lapho uke weva buhlungu khona emphilweni yakho?

Mental health:

1. When this happened and after it happened how did you feel?

Lekhatsi kwenteka loko, nangemuva kwako, wativa njani?

1. What other things were happening in your life around this time?

Ng’tiphi letinye tintfo lebetenteka emphilweni yakho ngalesos’khatsi?

1. What stories have you heard about difficult or hurtful times your parents have experienced? Uke weva ngebumatima nobe kuhlukunyetwa lokwenteka kubatali bakho?
2. Have there been other times when you felt really worried, scared or sad? Please tell me about those. (NOTE: repeat question multiple times) Kuke kwaba netikhatsi lapho wativa wesaba nobe ukhatsatekile nobe ukhubekile? Ngicela ungitjele kabanti ngaletotikhatsi.
3. Have there been times when you thought about hurting yourself or hurting others? Please tell me about those. Uke waba nemicabango yekutilimata nobe kulimata lomunye muntfu? Ngicela ungitjele ngayo lemicabango.
4. Tell me about times in your life when you felt very very angry. What happened? Ngitjele ngetikhatsi emphilweni yakho lapho wativa utfukutsele (ukwatile) kakhulu khona? Bekwentekeni noma kwentekani?

Parenting:

1. Tell me about your relationship with your child or children?

Ngitjele kabanti ngebudlelwane bakho nemtfwana nobe bantfwa bakho?

1. What are your biggest worries about your child or children?

Ng’tiphi tintfo letikukhatsata kakhulu ngemtfwana nobe bantfwa bakho?

1. What difficulties or hurtful things has your child or children experienced so far growing up? Ngubuphi bumatima nobe tintfo letihlukubetako, umntfwana wakho nobe bantfwana bakho lase bandlule kuko ekukhuleni kwabo?
2. What are your biggest regrets as a parent?

Kukhona yini tintfo lotisola ngato kakhulu wena njengemtali?

1. If you think about growing up, how was your relationship with your mother and father, and anyone else who raised you? (NOTE: does participant talk about biological parent or other parents? Who lived with?) Nawucabanga ngekukhula kwakho, bebunjani budlelwane bakho nebatali bakho, nanobe ngubani lo wakukhulisa?

HIV:

1. For those HIV-positive –
   1. We are really interested in understanding women’s experiences in living with HIV. Can you tell me about your experience? Sifuna kwati kabanti ngesimo salabasikati labaphila neligciwane leHIV. Ungangi tjela kabanti ngesimo sakho?

Thinking about when you first found out you have HIV, tell me about your journey in living with HIV.

- 1. Uma ukhumbula ngalesikhatsi ucala kuva kutsi unaleligciwane leHIV, ungangitjela kabati ngluhambo lwakho lekuphila nalo leligciwane?
     1. How have other people reacted to you?
     2. How has it affected your relationships with both parents and with your children and partner?

Loku kutsikabete kanjani budlelwane bakho nebatali bakho, nebantfwa bakho kanye nemlingani wakho?

- - 1. How has living with HIV affected your life?

Kuk’tsikamete njani imphilo yakho, kuphila naleli gciwane leHIV?

1. For those not living with HIV –
   1. HIV affects many people living around here. How has HIV affected you or your family? Please tell me about that. Ligciwane leHIV litsikameta bantfu labanyeti labahlala khona la. Wena nemndeni wakho, linitsikamete kanjani? Ungangitjela kabanti?

Closing

1. Thinking about your life, your parents’ life and your children’s life, tell me about the similarities and differences you see?

Uma ucabanga ngemphilo yakho, imphilo yebatali bakho nebantfwana bakho, ungangitjela ngetintfo letifanako netintfo letahlukile lotibonako?

- 1. What do you think are the reasons for the similarities?

Nawucabanga ng’tiphi tizatfu letenta kutsi kube netintfo letifanako?

- 1. What do you think are the reasons for the differences?

Nawucabanga ng’tiphi tizatfu letenta kutsi kube nemehluko?

1. If you look back at your drawing/sandbox, you’ve told me about times that have been difficult. Uma ubuka lesitfombe losidwebile, ungitjele ngetikhatsi lebetimatima.
   1. What helped you get through those times?

Yini leyakusita ek’tseni wendlule kuletotikhatsi?

- 1. What have you learned about yourself.

Yini loyifundze ngawe?

1. For option 3 (sandbox) –
   1. Looking at your tray now, is there anything that makes you feel uncomfortable that you’d like to change? Please go ahead and change your tray.

Uma ubuka indishi yakho, kukhona intfo lekwenta ungaphatseki kahle? Intfo mhlawumbe lofisa kuyintjintja? Khululeka untjintje lofisa kukuntjintja keyo lendishi yakho.

- 1. Please tell me about your tray. Ngicela ungitjele kabanti ngayo lendishi yakho.

Is there anything else you would like to tell me about the topics we have discussed today?

If not, that is the end of our discussion. Do you have any questions for me?

Thank you for your time and participation, it is really appreciated!

__________________________________________________________________________________

Before participant leaves, for those who have created pictures ask them:

What would you like to do with your drawings? (if the participant says they would like to take the drawings, make sure it is safe to do so. Ask if you can take a picture of the images before they leave. If participant says they would like to destroy the drawings, ask permission to take pictures first and let them destroy. If the participant chooses to leave the drawings, let them know you will take a picture of them and then store the image).

(Note to interviewers: To protect the safety of participants, it is *strongly* encouraged to not allow them to keep the drawing they create depicting any violence, as it could disclose to others what they talked about. Instead, collect the drawing showing violence but end the interview by creating one additional drawing with them. Ask them to draw “their happy place.” Engage with them and have fun as they create this drawing. Allow them to keep this drawing and take it home instead.)

For those who completed a sandbox, ask permission to take a photo of their first tray and then their second tray when there has been a change made.
