# Supplementary material for: “I’ve accepted it because at the end of the day there is nothing, I can do about it”: A qualitative study exploring the experiences of women living with the HIV, intimate partner violence and mental health syndemic in Mpumalanga, South Africa
Source: PLOS Glob Public Health. 2024 May 6;4(5):e0002588. doi: 10.1371/journal.pgph.0002588 (PMC11073682; doi:10.1371/journal.pgph.0002588)
Supplement: S2 Text — (DOCX) [file pgph.0002588.s003.docx]

**Supplementary File 3: Art-Based Methods Prompts**

**Squiggle drawing warm up:**

We are going to do a squiggle drawing together to get used to the materials here.

Give participant piece of paper and interviewer take piece of paper as well. Make all drawing materials available to participant. Encourage exploration and fun.

Please choose a marker or crayon you’d like and when I say ‘squiggle’ please go ahead and create a squiggle on the page. Take your time and just see what you come up with. There is not right or wrong way to do this.

If participant doesn’t understand, please demonstrate. Give direction to squiggle and let participant draw as long as they like. Interviewer does the same.

That’s great! Now, if you look at your squiggle drawing, is there anything you see in those lines? Take your time and look until something jumps out at you.

Encourage participant to turn page slowly and look from all angles/sides until and image or symbol jumps out. Interviewer does the same. Interviewer can demonstrate on their paper first if participant doesn’t understand.

When you see an image or symbol, please choose another marker or crayon and outline it so I can see it too. Remember there is no right or wrong way to do this!

Interviewer completes the same exercise. Encourage fun and participation.

Well done! I can see your image/symbol too…can you see mine? Now if this picture had a name, what would it be called?

Encourage participant to title the drawing and write the title on the page. Interviewer does the same.

**OPTION 1**

Kinetic Family Drawing (KFD):

Now, please can you draw some more pictures. Remember there is no right or wrong way to draw. The first picture will be about your family now (current family), and the next will be about the family you were born in. Would you please ***‘*draw a picture of everyone in your family, including you, doing something. Try to draw whole people, not cartoons or stick people. Remember, make everyone doing something, some kind of action.’**

Give participant one piece of paper and all the drawing materials. Remind them that the first KFD is about their current family. Give participants as much time as they need to complete. You may have to repeat the instruction exactly as it is written above again.

Once they are done with the first image, please give participants another piece of paper and remind them to complete a KFD about their family of origin. If the participant lived in many places or families growing up, ask them to focus on who they lived with for most of the time or who they lived with as adolescents. It may take some time for participants to remember and draw. You may have to repeat the instruction exactly as written above again.

Thank you. If each of these pictures had a name, what would they be called? Please write that on the page. Now, please can you tell me about your pictures.

Remember to be open to their story and to their images. Have a sense of curiosity about what is happening in the picture and what is happening between the people in the picture.

**OPTION 2: River of Life:**

Now, please can you draw another picture. Remember there is no right or wrong way to draw. Here is a piece of paper. Please imagine a river that shows your life from beginning to this point. Think about things that stand out for you in your life and you can draw these or write words to show important things. These may be good things or difficult things.

Give participant one piece of paper and all the drawing materials. Give participants as much time as they need to complete – it may take some time to remember past events and complete the task. You may have to repeat the instruction above again.

Thank you. If this picture had a name, what would it be called? Please write that on the page. Now, please can you tell me about your picture.

Remember to be open to their story and to their image. Have a sense of curiosity about what is happening in the picture and what memories have been incorporated.

**OPTION 3: Sandbox**

Now can we do an exercise with the sand tray please? Take a look at all these objects. Please select an object that represents you and also objects that represent important people in your life (both living and dead) and make a world in the sand or anything that comes to mind. Take your time and let me know when you are done.

Interviewers will offer participants the sandtray as well as the figures/objects and will invite participants to engage in the task. Participants will be given the opportunity to explore the figures and take their time placing these in the sand.

Thank you. If this tray scene had a name what would it be called? Now let’s look at the tray together. Please tell me about your tray.
