# Supplementary material for: “I’ve accepted it because at the end of the day there is nothing, I can do about it”: A qualitative study exploring the experiences of women living with the HIV, intimate partner violence and mental health syndemic in Mpumalanga, South Africa
Source: PLOS Glob Public Health. 2024 May 6;4(5):e0002588. doi: 10.1371/journal.pgph.0002588 (PMC11073682; doi:10.1371/journal.pgph.0002588)
Supplement: S1 Checklist — (DOCX) [file pgph.0002588.s004.docx]

STROBE Statement—checklist of items that should be included in reports of observational studies.

|  | Item No. | Recommendation | Page  No. | Relevant text from manuscript |
| --- | --- | --- | --- | --- |
| **Title and abstract** | 1 | (*a*) Indicate the study’s design with a commonly used term in the title or the abstract | 2 | *“I’ve accepted it because at the end of the day there is nothing, I can do about it”*: A qualitative study exploring the experiences of women living with the HIV, intimate partner violence and mental health syndemic in Mpumalanga, South Africa |
|  |  | (*b*) Provide in the abstract an informative and balanced summary of what was done and what was found | 3 | **Abstract**  In South Africa, Mental Health (MH), HIV, and Intimate Partner Violence (IPV) form a syndemic, that disproportionately affects women. These challenges are often co-occurring and create complex adversities for women. Recognising these intersections and the broader socio-cultural dynamics at play is crucial to understanding the layered experiences of these women and developing effective interventions. This research explores the experiences of the women living with at least two of the epidemics (HIV, IPV and or MH) and how they cope. A qualitative study design was used and 20 women (22-60 years) were recruited from Mpumalanga, South Africa. To be eligible for the study the women had to have experienced at least two of the epidemics. Data were collected through home-based interviews, arts-based activities, and analysed thematically using MAXQDA (2022) software. MH challenges were prevalent among all the participants and were linked to both IPV and HIV, resulting in symptoms such as anxiety, depression, and suicidal thoughts. In relation to the HIV-MH link, MH challenges in this combination included feelings of denial, sadness and anxiety related to participant’s HIV diagnosis. A bidirectional relationship also existed in the IPV-MH group where pre-existing MH challenges among women increased their vulnerability of having violent partners, whilst IPV also increased MH challenges. In the IPV-MH-HIV group early childhood violence exposure was linked with MH challenges and later victimization and vulnerability to HIV. Participants primarily used religion, acceptance, occasional alcohol, and family support as coping strategies. Particularly in IPV situations, alcohol use/misuse was the most prevalent coping strategies. The study highlights the syndemic relationship between HIV, IPV and MH challenges among South African women living in a peri-urban community, with a central emphasis on MH challenges. Interventions should holistically address these challenges, with particular focus on MH challenges, cultural sensitivity, and promotion of healthy coping strategies. |
| Introduction | | | |  |
| Background/rationale | 2 | Explain the scientific background and rationale for the investigation being reported | 4-7 |  |
| Objectives | 3 | State specific objectives, including any prespecified hypotheses | 7 | The primary objective of the present study was to explore the experiences of women navigating life with the intersections of HIV, IPV and MH challenges in a peri-urban community in Mpumalanga South Africa. We aimed to analyse the interplay of the epidemics and better understand the coping strategies used by the women. Furthermore, we sought to identify variations in coping strategies based on the different combinations of the epidemics experienced by the women. |
| Methods | | | |  |
| Study design | 4 | Present key elements of study design early in the paper | 7-12 |  |
| Setting | 5 | Describe the setting, locations, and relevant dates, including periods of recruitment, exposure, follow-up, and data collection | 7 | The current study was conducted in a peri-urban area within the Enhlanzeni District Municipality of Mpumalanga Province, South Africa from October 2022 to February 2023. |
| Participants | 6 | (*a*) *Cohort study*—Give the eligibility criteria, and the sources and methods of selection of participants. Describe methods of follow-up  *Case-control study*—Give the eligibility criteria, and the sources and methods of case ascertainment and control selection. Give the rationale for the choice of cases and controls  *Cross-sectional study*—Give the eligibility criteria, and the sources and methods of selection of participants | 7 | 1. Twenty participants were purposively selected for the study. Selection of the participants was based on their responses to specific questionnaire items from the Interrupt_Violence Study (34). Participants were between the ages of 20-60 years and had to screen positive for HIV, IPV and/or MH challenges. Participants did not have to have experienced all three epidemics to be included in the study; any combination of the two epidemics was sufficient for inclusion. |
|  |  | (*b*) *Cohort study*—For matched studies, give matching criteria and number of exposed and unexposed  *Case-control study*—For matched studies, give matching criteria and the number of controls per case |  |  |
| Variables | 7 | Clearly define all outcomes, exposures, predictors, potential confounders, and effect modifiers. Give diagnostic criteria, if applicable |  | N/A |
| Data sources/ measurement | 8* | For each variable of interest, give sources of data and details of methods of assessment (measurement). Describe comparability of assessment methods if there is more than one group | 14 | The lead author alongside two research assistants conducted in-depth interviews which lasted between 60 to 90 minutes with each participant during October 2022-February 2023. The interviews were conducted in the participants’ preferred languages of SiSwati or Xitsonga and took place face-to-face in a private space at a time convenient for the participants. The interviews allowed for probing into participants’ experiences with IPV, living with HIV, and navigating MH challenges. We explored their past and current experiences with IPV, the impacts of HIV diagnosis and its disclosure, and the community’s potential stigma towards HIV. Furthermore, their MH journey was explored in the context of these intersecting epidemics.  To stimulate rich conversation, we used arts-based techniques such as the Kinetic Family Drawing (KFD), River of Life, and Sandbox. |
| Bias | 9 | Describe any efforts to address potential sources of bias | 31-32 | Another limitation concerns the method used for categorizing participants into different epidemic combinations. While this approach initially provided a structured framework for analysis, it might have introduced some degree of arbitrariness, where in some cases some participants may not have met the criteria of the questionnaire leading them to not being categorized as experiencing a particular MH challenge. However, to refine this categorization, we re-categorized the groups, based on the information gathered from the qualitative interviews. For example, participants not initially flagged for MH challenges in the questionnaire but who later revealed symptoms indicative of such during the interviews were re-categorized accordingly. This additional layer of categorization aimed to capture more accurately the complexities of each participant's experiences.  We acknowledge that our personal beliefs, cultural understanding, and personal experiences may have shaped our interpretation as well as the presentation of the participants’ stories and as a result potentially introducing subjective biases. As an attempt to mitigate this, the research team had regular debriefing sessions throughout the study, where we discussed and challenged each other’s viewpoints, which helped in ensuring a more balanced perspective on the data. Furthermore, the interviewers also kept detailed field notes after the interviews which were reflective accounts of the interviews including the interviewer thoughts and reactions to the interview. |
| Study size | 10 | Explain how the study size was arrived at |  |  |

Continued on next page

| Quantitative variables | 11 | Explain how quantitative variables were handled in the analyses. If applicable, describe which groupings were chosen and why |  |  |
| --- | --- | --- | --- | --- |
| Statistical methods | 12 | (*a*) Describe all statistical methods, including those used to control for confounding |  |  |
|  |  | (*b*) Describe any methods used to examine subgroups and interactions |  |  |
|  |  | (*c*) Explain how missing data were addressed |  |  |
|  |  | (*d*) *Cohort study*—If applicable, explain how loss to follow-up was addressed  *Case-control study*—If applicable, explain how matching of cases and controls was addressed  *Cross-sectional study*—If applicable, describe analytical methods taking account of sampling strategy |  |  |
|  |  | (*e*) Describe any sensitivity analyses |  |  |
| Results | | | | |
| Participants | 13* | (a) Report numbers of individuals at each stage of study—eg numbers potentially eligible, examined for eligibility, confirmed eligible, included in the study, completing follow-up, and analysed |  |  |
|  |  | (b) Give reasons for non-participation at each stage |  |  |
|  |  | (c) Consider use of a flow diagram |  |  |
| Descriptive data | 14* | (a) Give characteristics of study participants (eg demographic, clinical, social) and information on exposures and potential confounders |  |  |
|  |  | (b) Indicate number of participants with missing data for each variable of interest |  |  |
|  |  | (c) *Cohort study*—Summarise follow-up time (eg, average and total amount) |  |  |
| Outcome data | 15* | *Cohort study*—Report numbers of outcome events or summary measures over time |  |  |
|  |  | *Case-control study—*Report numbers in each exposure category, or summary measures of exposure |  |  |
|  |  | *Cross-sectional study—*Report numbers of outcome events or summary measures |  |  |
| Main results | 16 | (*a*) Give unadjusted estimates and, if applicable, confounder-adjusted estimates and their precision (eg, 95% confidence interval). Make clear which confounders were adjusted for and why they were included |  |  |
|  |  | (*b*) Report category boundaries when continuous variables were categorized |  |  |
|  |  | (*c*) If relevant, consider translating estimates of relative risk into absolute risk for a meaningful time period |  |  |

Continued on next page

| Other analyses | 17 | Report other analyses done—eg analyses of subgroups and interactions, and sensitivity analyses | 14-15 | First, four researchers reviewed and assessed at least three transcripts each of interviewer-led qualitative interviews, to appraise pilot processes and application, giving feedback to interviewers on utilization of methods, interaction with participants and quality of data gathered. Researchers discussed reflections of the transcripts reviewed (that were grouped into themes by the first author) and together with observations from the field and team meeting discussion over a 5 month period, triangulation primed inclusive discussion guides for the FGDs that followed (49). Deliberations on central findings after every FGD was concluded with the broader investigative team ensured nuanced questions were incorporated in the next round of discussions, guaranteeing all areas of investigation were being grasped and studied. A rapid analytic approach (50,51) was used to analyse data. This swift and iterative approach to data collection and analysis was undertaken to understand the pilot study, including the acceptability, feasibility and appropriateness of the study methods and materials, and chosen for its capacity to produce targeted research in a timely way to inform the main study tools and implementation that was forthcoming (52). Study investigators who analysed the data were embedded in the study with operational understanding of the context and methods, offering insight to the complexity of the study implementation. The analysis was minimally interpretive with the first author establishing an inventory of the data contents or main domains, derived from the interview and discussion guides, condensing and consolidating the data into summaries (53). Three researchers read all FGD transcripts, underscoring significant quotes that matched the inventory generated, and clarified or confirmed summarized significant findings collectively. |
| --- | --- | --- | --- | --- |
| Discussion | | | | |
| Key results | 18 | Summarise key results with reference to study objectives | 22 | Our pilot study found that using arts and play-based methods in multigenerational violence research is feasible and acceptable to participants and interviewers. These methods worked well for nearly all participants regardless of age or ability. They can enhance what researchers (and participants) can discover through their capacity to facilitate disclosure, particularly around stigmatizing and sensitive experiences like violence (16). Perhaps this is due to the increased safety these methods provide in their ability to facilitate expression and allow participants personal distance from the content of violence they are sharing with interviewers, enhancing critical thinking (54) Our study appeared to support other local research with vulnerable violence exposed participants and their perceived benefit of inclusion, having an opportunity to share adverse experiences with others in a ‘safe space’ (55) |
| Limitations | 19 | Discuss limitations of the study, taking into account sources of potential bias or imprecision. Discuss both direction and magnitude of any potential bias | 29 | Rapid data analysis helped identify the context specific issues that needed to be addressed and understood (both on the part of participants but also interviewers) related to the data collection tools utilized for the pilot. However, rapid research designs tend to use small sample sizes which complicates generalizability of findings. Also, this analysis strategy did not benefit from a deeper thematic analysis of data.  Although interviewers where trained and mentored in the methods used, there was a limitation in deepening interviews and facilitating more probing around significant participant responses. As a result, the main study employed more experienced qualitative researchers. Participants may also have been biased through social desirability. |
| Interpretation | 20 | Give a cautious overall interpretation of results considering objectives, limitations, multiplicity of analyses, results from similar studies, and other relevant evidence | 22-29 |  |
| Generalisability | 21 | Discuss the generalisability (external validity) of the study results |  |  |
| Other information | |  | | |
| Funding | 22 | Give the source of funding and the role of the funders for the present study and, if applicable, for the original study on which the present article is based |  | *Funding This study is funded by the European Research Council (ERC) under the European Union’s Horizon 2020 research and innovation programme (Grant Agreement Number 852787) and the UK*  *Research and Innovation Global Challenges Research Fund (ES/S008101/1).* |

*Give information separately for cases and controls in case-control studies and, if applicable, for exposed and unexposed groups in cohort and cross-sectional studies.

**Note:** An Explanation and Elaboration article discusses each checklist item and gives methodological background and published examples of transparent reporting. The STROBE checklist is best used in conjunction with this article (freely available on the Web sites of PLoS Medicine at http://www.plosmedicine.org/, Annals of Internal Medicine at http://www.annals.org/, and Epidemiology at http://www.epidem.com/). Information on the STROBE Initiative is available at www.strobe-statement.org.
